# Quantitative Succinyl-Proteome Profiling of *Camellia sinensis* cv. ‘Anji Baicha’ During Periodic Albinism

**DOI:** 10.1038/s41598-017-02128-x

**Published:** 2017-05-12

**Authors:** Yan-Xia Xu, Chen-Jia Shen, Jian-Qiang Ma, Wei Chen, Juan Mao, Yan-Yan Zhou, Liang Chen

**Affiliations:** 1 Tea Research Institute of the Chinese Academy of Agricultural Sciences/Key Laboratory of Tea Biology and Resources Utilization, Ministry of Agriculture, 9 South Meiling Road, Hangzhou, 310008 China; 2 College of Life and Environmental Sciences, Hangzhou Normal University, Hangzhou, 310036 China; 3 Jingjie PTM Biolab (Hangzhou) Co., Ltd., Hangzhou, 310018 China

## Abstract

Lysine succinylation is a novel dynamic and evolutionarily conserved post-translational modification (PTM) that regulates various biological processes. ‘Anji Baicha’ is an albino tea variety that exhibits temperature-based variability of leaf colour and amino acid concentrations. However, the mechanism underlying albinism in ‘Anji Baicha’ has not been investigated at the level of succinylation. Here, we identify 3530 lysine succinylation sites mapped to 2132 proteins in ‘Anji Baicha’, representing the first extensive data on the lysine succinylome in the tea plant. Eleven conserved succinylation motifs were enriched among the identified succinylated peptides. The protein-protein interaction maps were visualized using Cytoscape software. Comparison across three typical developmental stages of ‘Anji Baicha’ revealed that proteins exhibiting differential succinylation levels were primarily involved in photosynthesis, carbon fixation, biosynthesis of amino acids and porphyrin and chlorophyll metabolism, suggesting that these succinylated proteins are involved in ‘Anji Baicha’ leaf colour variability. These results not only deepen our understanding of the mechanism underlying ‘Anji Baicha’ albinism and the regulatory role of succinylation in the tea plant but also provide new insight into molecular breeding for leaf colour variety.

## Introduction

Post-translational modification (PTM) plays important roles in many cellular physiological processes, including cell cycle regulation and apoptosis; cell morphology; cell differentiation; metabolic pathways; protein interactions; and enzymatic activity^1^. There are more than 400 types of PTMs, including methylation, phosphorylation, ubiquitination, acetylation and succinylation, and mounting evidence has indicated that they are important mechanisms by which protein function is expanded^2, 3^. Among all PTMs, succinylation is one of the PTMs that being the most recently identified and the least well characterized.

The existence of lysine succinylation has been suggested in previous studies^4–6^. However, these studies did not conclusively determine the chemical structure of the modification. In 2011, lysine succinylation was first identified as a PTM by Zhang *et al*.^7^, who reported that lysine succinylation is a dynamic and reversible process that transfers succinyl group (-CO-CH_2_-CH_2_-CO-) from succinyl-coenzyme A (succinyl-CoA) to the ε-amino group of lysine residue of the target protein, thereby changing the structure and function of specific proteins under various physiological conditions^7–9^. The mitochondrial protein sirtuin, or SIRT5, is a potent desuccinylase^10^. Recently, high-resolution mass spectrometry (MS) and antibody-based affinity enrichment of succinylated lysine residues has provided researchers with an opportunity to identify a large number of lysine-succinylated proteins and relevant sites in bacteria^7, 9, 11^, yeast^8, 9^, protozoa^12^, animals^8–10, 13^ and humans^8, 9^, indicating that lysine succinylation is widespread and evolutionarily conserved among prokaryotes and eukaryotes^9, 11^ and this PTM has diverse cellular functions. Succinylation occurs at many sites and exhibits crosstalk with other PTMs, including acetylation^9^. Some researchers have found evidence that lysine succinylation impacts mitochondrial metabolism and coordinates different metabolic pathways at the PTM level^13–17^. In plants, little was known about lysine succinylation until He *et al*.^3^ and Shen *et al*.^18^ identified an abundance of succinylation sites (SSs) and succinylation proteins (SPs) in rice and *Taxus*, respectively.

Tea [*Camellia sinensis* (L.) O. Kuntze], one of the most important cash crops in the world, is a non-alcoholic beverage that greatly benefits human health^19^. Tea plants cultivated in Asia account for approximately eighty percent of the world’s tea production. ‘Anji Baicha’ (alias ‘Baiye 1’ or ‘White Leaf 1’), a special green tea cultivar widely grown in China, has attracted the attention of researchers on account of its unique characteristics of temperature-based leaf colour variability and amino acid concentrations^20–25^. When the temperature is below 20 °C in early spring, chlorina gradually occurs in leaves that are yellow-green in the pre-albinistic stage and white in the albinistic stage, followed by normal green in the re-greening stage when the temperature rises above 22 °C^20–22^. Accompanied with colour changes, chloroplast ultrastructure and chemical composition gradually changes during periodic albinism. One possible explanation of this phenomenon is that exposure to low temperatures during the bud-emerging stage in early spring could reduce the number and disrupt the structure of chloroplasts, leading to the decreased chlorophyll content exhibited by the albino phenotype. As temperatures rise, the number and structure of chloroplasts would then gradually recover, resulting in increased chlorophyll content and the return of green leaves^22^. Interestingly, the amino acid content—particularly that of theanine—exhibits a change opposing that of chlorophyll, whereas the tea polyphenol content changes in a similar manner to that of chlorophyll^23–25^. In addition, the enzyme activity of these plants was investigated, revealing that peroxidase and proteinase activities were enhanced, whereas superoxide dismutase, catalase and rubisco activities were reduced during the albinistic stage^26, 27^. Recently, studies have been conducted at the molecular level. Ma *et al*.^28^ and Yuan *et al*.^29^ studied the differential expression of genes during periodic albinism via cDNA microarray and amplified fragment length polymorphism (AFLP) techniques, respectively. Xiong *et al*.^25^ and Wu *et al*.^30^ analysed catechin biosynthesis-related gene expression by quantitative real-time PCR and transcriptome characterization, respectively. Li *et al*. reported differential metabolic profiles during periodic albinism^31^. Using two-dimensional electrophoresis and MS, Li *et al*. investigated the differential expression of proteins at three typical developmental stages^22^. However, protein expression data remain lacking, especially with respect to protein modification, and the molecular mechanism underlying the physiological processes in ‘Anji Baicha’ remains unknown. Here, we report the first proteomics-based quantification of lysine succinylation in *C*. *sinensis* cv. ‘Anji Baicha’ leaves during periodic albinism. This study provides new insight into the molecular mechanism underlying leaf colour change in ‘Anji Baicha’ at the PTM level.

## Results

### Changes in colour and chlorophyll concentration in ‘Anji Baicha’ during periodic albinism

**Figure 1:**
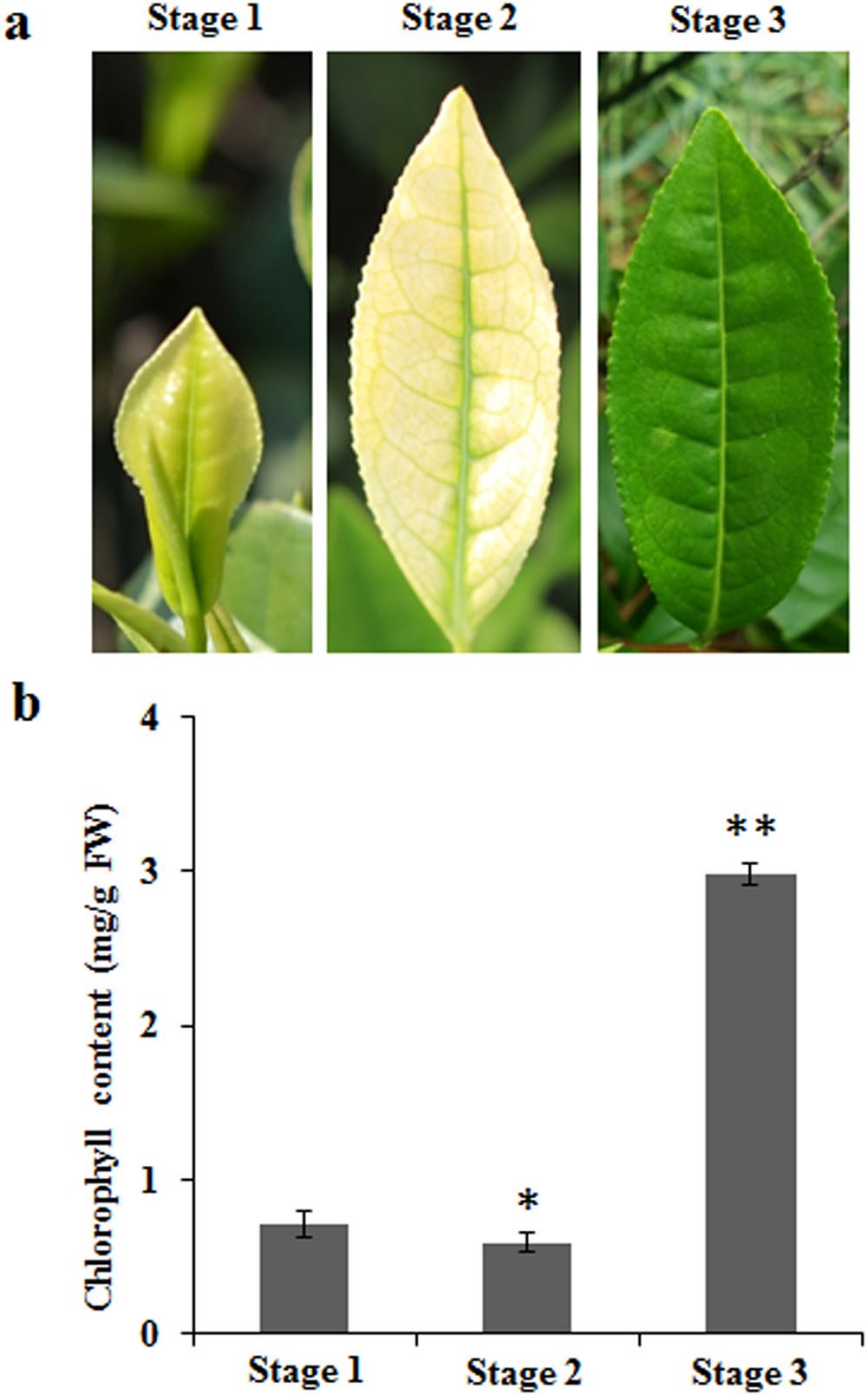
‘Anji Baicha’ leaves and chlorophyll concentrations during three developmental stages. (**a**) ‘Anji Baicha’ leaves. (**b**) Chlorophyll concentrations. All experiments were analysed using five independent biological replicates. *Indicates a significant difference at P < 0.05, and **Indicates a significant difference at P < 0.01 (Student’s t test).

‘Anji Baicha’ is a temperature sensitive albino tea variety that is widely grown in China. Here, we divided ‘Anji Baicha’ leaves into three typical developmental stages based on the characteristics of periodic albinism: the pre-albinistic stage (Stage 1), the albinistic stage (Stage 2) and the re-greening stage (Stage 3) (Fig. 1a). The chlorophyll concentrations of ‘Anji Baicha’ during the three stages were measured (Fig. 1b). We found that the chlorophyll concentration during Stage 2 was the lowest, and the chlorophyll concentration in Stage 3 was significantly higher than that of Stage 1 and Stage 2 (4-fold higher than Stage 1 and 3-fold higher than Stage 2). These results are in agreement with previous reports^20, 21^.

### SP identification and SS localization

**Figure 2:**
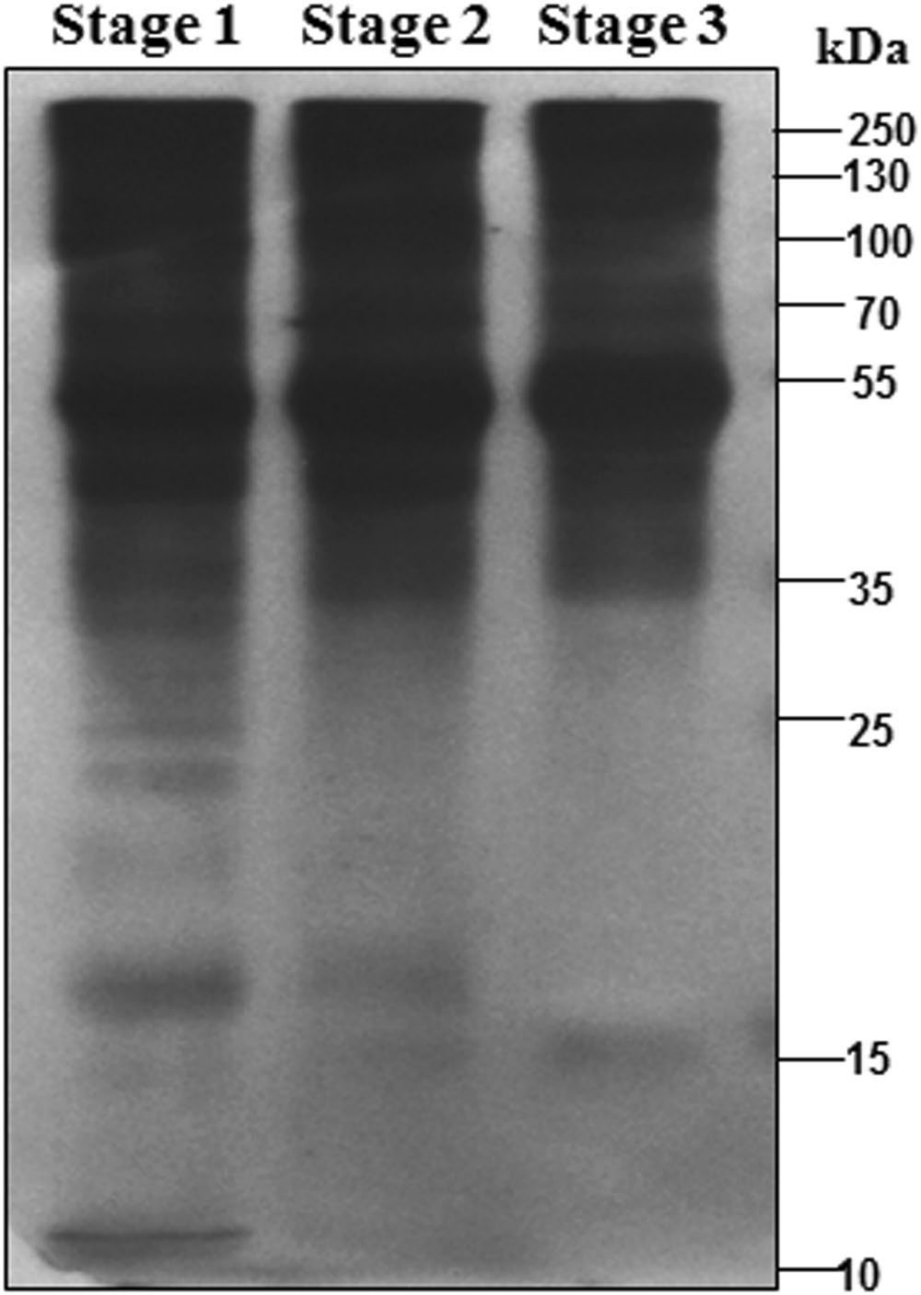
Western blotting analysis of the succinylation levels among the three ‘Anji Baicha’ developmental stages.

Lysine succinylation is evolutionarily conserved and highly dynamic and plays an important role in various cellular and developmental processes in both prokaryotic and eukaryotic cells^9, 11^. To date, the succinylome of the tea plant has not been reported. To identify the presence of SPs in tea plant, western blotting analysis was performed using an anti-succinyl lysine antibody. The results showed that a lot of proteins with a wide range of molecular masses are succinylated (Fig. 2). The immunoblot signals were strong, suggesting that lysine succinylation was abundant in tea plant (Fig. 2). Using tandem mass tag (TMT) labelling and affinity enrichment followed by high-resolution liquid chromatography-tandem mass spectrometry (LC-MS/MS) analysis, we identified 3530 lysine SSs in ‘Anji Baicha’ leaves (see Supplementary Table S1) that exhibited distinct abundances depending on length (see Supplementary Fig. S1a). The 3530 lysine SSs were mapped to 2132 SPs for which between 1 and 13 SSs were identified (see Supplementary Table S1). Notably, luminal-binding protein 5 (CL8488.Contig1_All) processed 13 SSs, representing the most frequently occurring lysine-SP identified in this study. Two heat shock protein (CL1393.Contig10_All and CL1822.Contig7_All) had 12 SSs (see Supplementary Table S1).

**Figure 3:**
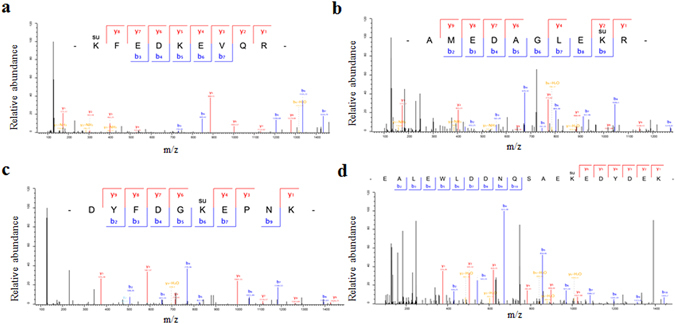
Four representative LC-MS/MS spectra of succinylated luminal-binding protein 5 (CL8488.Contig1_All). (**a**) Succinylated peptide K(su) FEDKEVQR, succinylation site at K109. (**b**) Succinylated peptide AMEDAGLEK(su)R, succinylation site at K360. (**c**) Succinylated peptide DYFDGK(su) EPNK, succinylation site at K389. (**d**) Succinylated peptide EALEWLDDNQSAEK(su) EDYDEK, succinylation site at K622.

Our MS data validation was evaluated by determining the mass error, which was approximately zero, and nearly all of the values were <5 ppm in three biological replicates, indicating that the MS data were highly accurate (see Supplementary Fig. S2). In addition, most peptides were between 7 and 20 amino acid residues long, indicating that the sample preparation met the technical standard (see Supplementary Fig. S1). Four representative succinylated luminal-binding protein 5 (CL8488.Contig1_All) LC-MS/MS spectra are shown in Fig. 3.

### Analysis of succinylated lysine motifs

**Figure 4:**
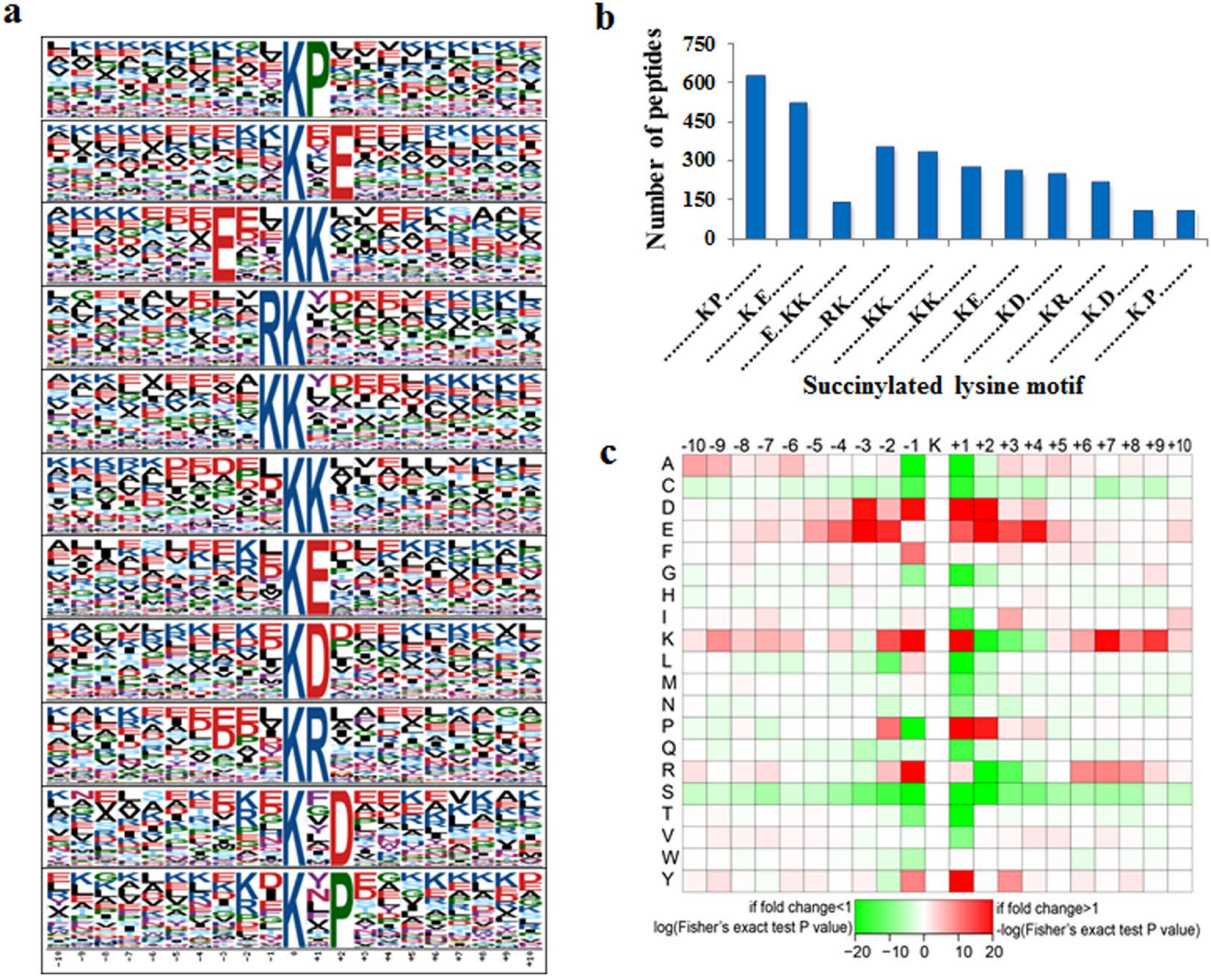
Motif-X analysis of the amino acids (±10) surrounding the identified succinylated residues. (**a**) Sequence logo representation of 10 conserved succinylation motifs. (**b**) The number of identified peptides possessing a succinylated lysine within each motif. (**c**) A plot showing the relative abundance of amino acids flanking a succinylated lysine, which is shown using the intensity map.

To evaluate the nature of the succinylated lysine residues present in ‘Anji Baicha’, we employ Motif-X to search for the sequence motifs. The results showed that 11 conserved succinylation motifs were enriched among all identified succinylated peptides, according to the criteria of a specific amino acid sequence spanning 10 amino acids upstream and downstream of the succinylated lysine (Fig. 4a). These 11 conserved succinylation motifs are ‘……….K^su^P………’, ‘……….K^su^.E……..’, ‘…….E..K^su^K………’, ‘………RK^su^……….’, ‘………KK^su^……….’, ‘……….K^su^K………’, ‘……….K^su^E………’, ‘……….K^su^D………’, ‘……….K^su^R………’, ‘……….K^su^.D……..’ and ‘……….K^su^.P……..’ (Fig. 4, Supplementary Table S2). As shown in Fig. 4b, of the 3530 identified lysine succinylation peptides, 3198 had above 11 conserved motifs, accounting for approximately 91% of all peptides. In addition, different conserved motifs exhibited different abundances; the motif ‘……….K^su^P………’ was the most abundant.

We generated a position-specific heat map to assess the preference for specific amino acids in specific positions (Fig. 4c). We observed a strong preference for aspartic acid (D), glutamic acid (E), and proline (P) in the +1 and +2 positions, as well as for lysine (K) and arginine (R) in the −1 position. Interestingly, the preference for D and E at +1 and +2 is consistent with the data reported in previous studies of other species, including *Escherichia coli* BW25113 and DH10B, as well as *Mycobacterium tuberculosis* H37Rv^32^, suggesting that the preference for succinylation 1 or 2 positions downstream of D and E is conserved. However, amino acid and positional preferences differ among species.

### Differences in lysine succinylation between three ‘Anji Baicha’ developmental stages

Western blotting analysis with the anti-succinyl lysine antibody showed that different developmental stages of ‘Anji Baicha’ may generate different succinylation profile (Fig. 2). In order to further investigate the differences in lysine succinylation between three ‘Anji Baicha’ developmental stages, we generated a proteome profiling and a quantitative succinylome profiling (Supplementary Tables S1 and S3). After succinylation changes were normalized against protein expression levels, we identified significant differences between three ‘Anji Baicha’ developmental stages. Compared to stage 1, 27 SSs in 25 SPs/18 SSs in 17 SPs were up-/down-regulated more than 1.5-fold in stage 2, and 139 SSs in 121 SPs/71 SSs in 65 SPs were up-/down-regulated more than 1.5-fold in stage 3, respectively. Furthermore, 82 SSs in 75 SPs/43 SSs in 42 SPs were up-/down-regulated more than 1.5-fold in stage 3 compared to stage 2 (see Supplementary Fig. S3 and Table S1).

**Figure 5:**
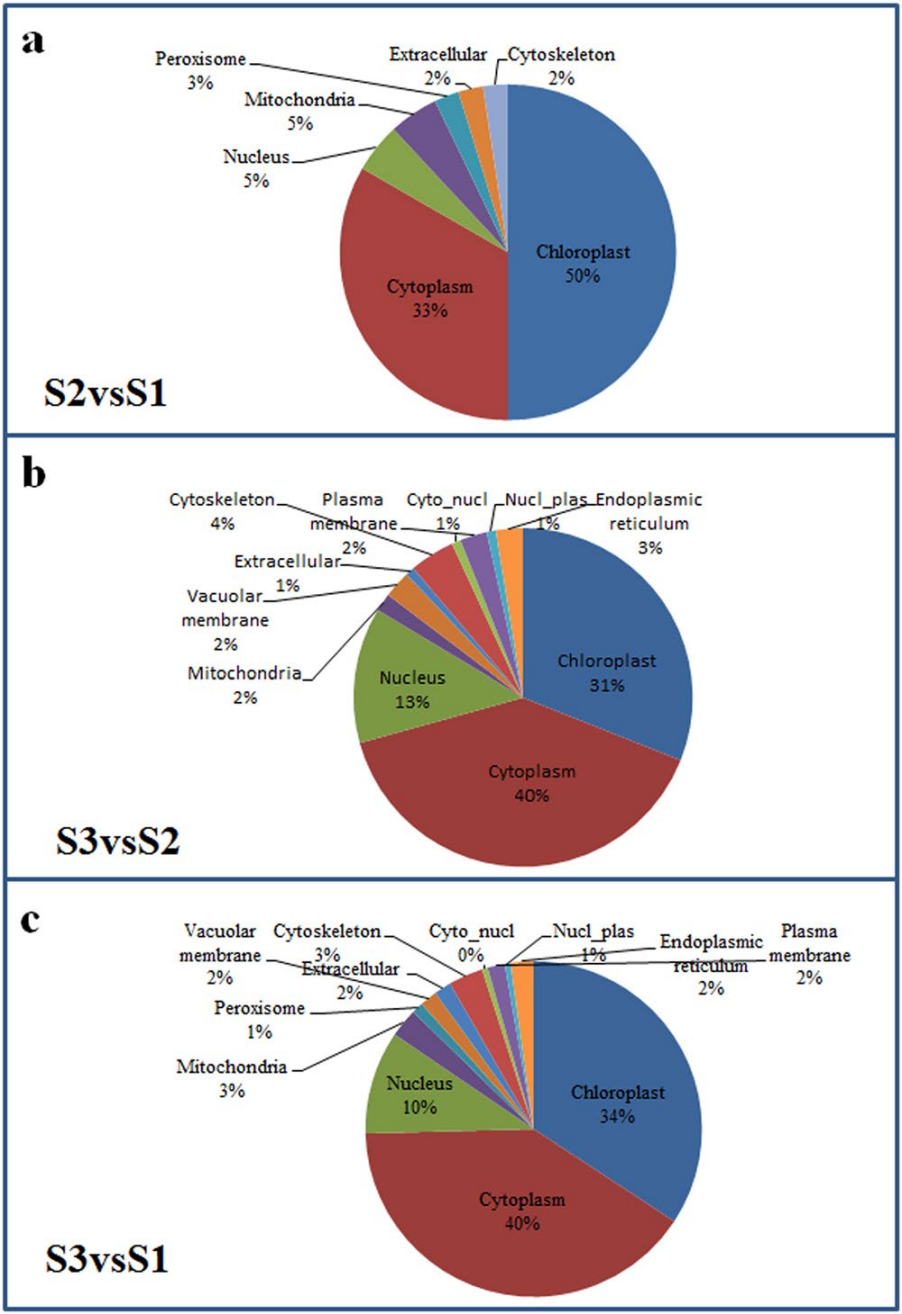
Subcellular location of PDSLs among the three ‘Anji Baicha’ developmental stages. (**a**) S2vsS1. (**b**) S3vsS2. (**c**) S3vsS1.

Few studies of the subcellular localization of succinyl-proteins have been reported. Therefore, we also analysed and classified the subcellular distribution of the proteins exhibiting differential succinylation levels (PDSL) (Fig. 5). In S2vsS1, 50% (21/42) of PDSLs are located in chloroplasts, 33% (14/42) in the cytosol, and 5% (2/42) in the nucleus (Fig. 5a). In S3vsS2, 31% (36/116) of PDSLs are located in chloroplasts, 40% (46/116) in the cytosol, and 13% (15/116) in the nucleus (Fig. 5b). In S3vsS1, 34% (62/181) of PDSLs are located in chloroplasts, 40% (73/181) in the cytosol, and 10% (15/181) in the nucleus (Fig. 5c). These results indicated that PDSLs are mainly located in chloroplasts and cytosol.

### Kyoto Encyclopedia of Genes and Genomes (KEGG) pathway analysis of PDSLs in three ‘Anji Baicha’ developmental stages

**Figure 6:**
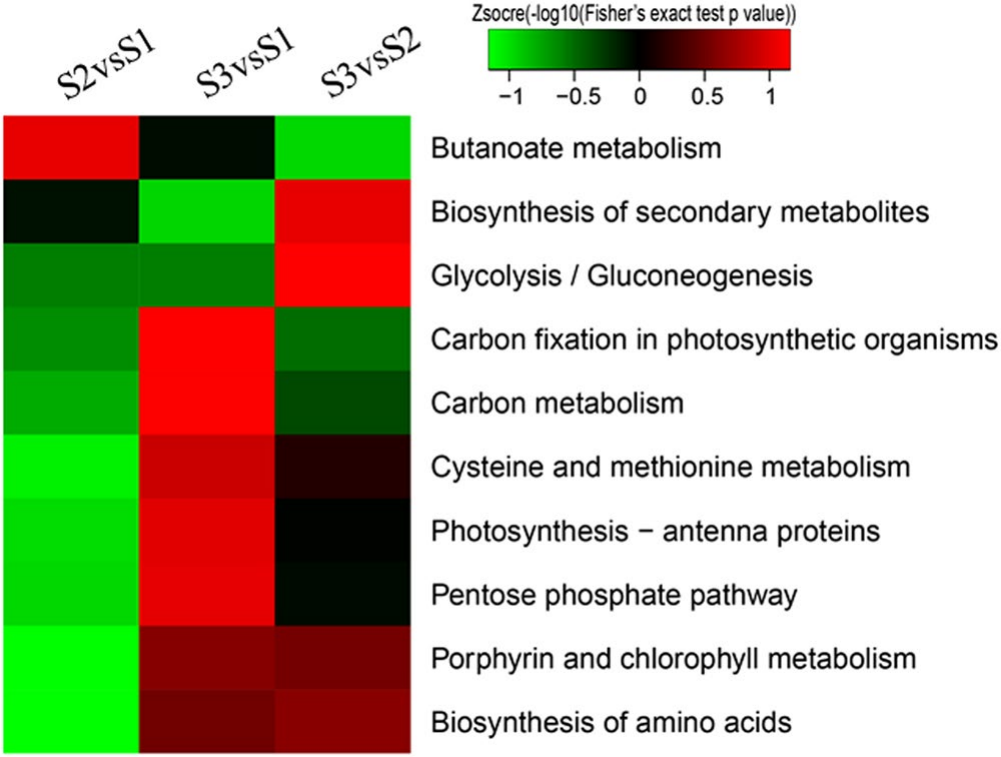
KEGG analysis of PDSLs among the three ‘Anji Baicha’ developmental stages.

To reveal PDSL pathways, KEGG pathway analysis was conducted. As shown in Fig. 6, butanoate metabolism was the main pathway in S2vsS1, whereas biosynthesis of secondary metabolites, glycolysis/gluconeogenesis, porphyrin and chlorophyll metabolism and biosynthesis of amino acids were the highlighted pathways in S3vsS2. In addition, photosynthesis and carbon fixation in photosynthetic organisms, carbon metabolism, pentose phosphate pathway, porphyrin and chlorophyll metabolism and biosynthesis of amino acids were the primary enriched pathways in S3vsS1. Notably, the most prominent pathways in S2vsS1 were different from those in S3vsS2 and S3vsS1, suggesting that the succinylation of proteins acting in specific pathways plays a critical role in specific ‘Anji Baicha’ developmental stages.

**Figure 7:**
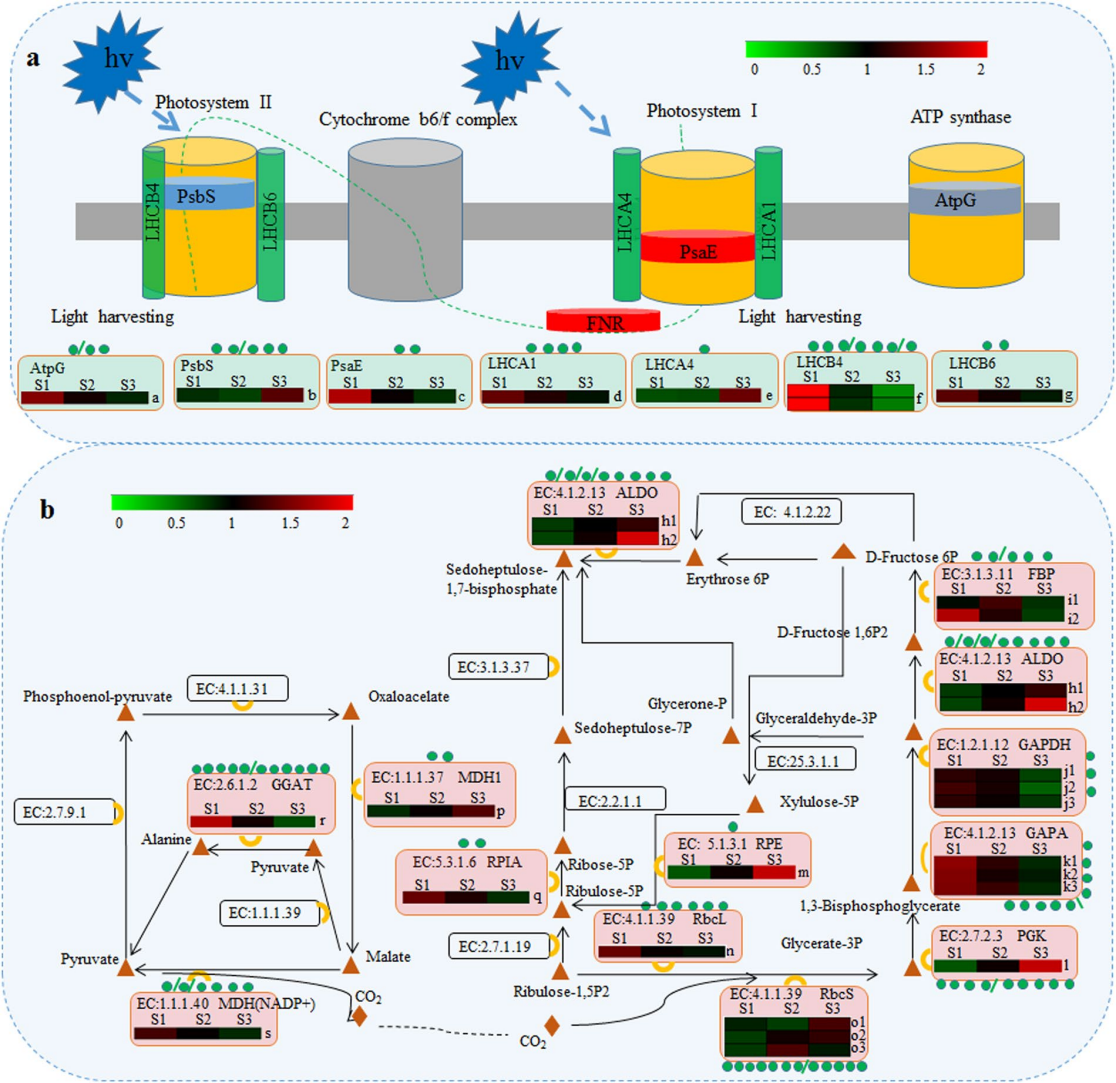
Differentially expressed SSs and SPs involved in photosynthesis and carbon fixation. The number of SSs is indicated by green dots. The succinylation level of differentially expressed SSs during the three ‘Anji Baicha’ developmental stages is shown using a heatmap. PsaE: photosystem I subunit IV; PsbS: photosystem II 22 kDa protein; LHCA1/4: light-harvesting complex I chlorophyll a/b binding protein 1/4; LHCB4/6: light-harvesting complex II chlorophyll a/b binding protein 4/6; AtpG: ATP synthase gamma chain; ALDO: fructose-bisphosphate aldolase, class I; FBP: fructose-1,6-bisphosphatase I; GAPDH/GAPA: glyceraldehyde 3-phosphate dehydrogenase/NADP+; GGAT: glutamate–glyoxylate aminotransferase; MDH: malate dehydrogenase; PGK: phosphoglycerate kinase; RbcL/S: ribulose-bisphosphate carboxylase large chain/small chain; RPE: ribulose-phosphate 3-epimerase; RPIA: ribose 5-phosphate isomerase A. a: Unigene17303_All, K89; b: CL2225.Contig1_All, K69; c: Unigene24422_All, K86; d: CL6420.Contig2_All, K98; e: CL8782.Contig1_All, K201; f: CL2645.Contig3_All, K209; g: Unigene3268_All, K56; h1: Unigene22463_All, K387; h2: Unigene48_All, K225; i1: CL1597.Contig1_All, K234; i2: CL509.Contig2_All, K353; j1: CL2420.Contig4_All, K120; j2: CL2420.Contig4_All, K143; j3: CL2420.Contig4_All, K254; k1: CL4565.Contig2_All, K159; k2: CL4565.Contig2_All, K205; k3: CL4565.Contig2_All, K341; l: Unigene21236_All, K125; m: CL2805.Contig4_All, K275; n: Unigene3523_All, K67; o1: Unigene9029_All, K144; o2: CL3466.Contig1_All, K67; o3: CL3466.Contig1_All, K148; p: CL6967.Contig2_All, K208; q: Unigene22450_All, K214; r: CL1792.Contig2_All, K463.

We further summarized the SSs and SPs involved in photosynthesis and carbon fixation in photosynthetic pathways (Supplementary Table S4). In total, 31 proteins related to photosynthesis were identified as SPs, including photosystem I P700 chlorophyll a apoprotein A2 (PsaB), photosystem I subunit II (PsaD), III (PsaF), IV (PsaE), X (PsaK), XI (PsaL) and N (PsaN), photosystem II CP43 chlorophyll apoprotein (PsbC), photosystem II oxygen-evolving enhancer protein 1 (PsbO), 2 (PsbP) and 3 (PsbQ), photosystem II 22 kDa protein (PsbS), photosystem II Psb27 protein (Psb27), light-harvesting complex I chlorophyll a/b binding protein 1 (LHCA1), LHCA2, LHCA4, light-harvesting complex II chlorophyll a/b binding protein 4 (LHCB4), LHCB5, LHCB6, ferredoxin NADP reductase (PetH), ATP synthase b chain (AtpB), delta chain (AtpD) and gamma chain (AtpG). Sixty-six lysine SSs were identified in these proteins, and many quantitative SSs were significantly different among the three ‘Anji Baicha’ developmental stages (Fig. 7a), suggesting that the succinylation of these above proteins may be associated with the periodic albino phenotype of ‘Anji Baicha’.

In the carbon fixation pathway, 102 lysine SSs were identified and mapped to fructose-bisphosphate aldolase (ALDO), fructose-1,6-bisphosphatase I (FBP), glyceraldehyde 3-phosphate dehydrogenase (GAPDH), glutamate–glyoxylate aminotransferase (GGAT), malate dehydrogenase (MDH), phosphoglycerate kinase (PGK), ribulose-bisphosphate carboxylase (Rbc), triosephosphate isomerase (TIM), transketolase (TKL), ribulose-phosphate 3-epimerase (RPE), phosphoenolpyruvate carboxylase (PPC), phosphoribulokinase (PRK), alanine transaminase (GPT), ribose 5-phosphate isomerase A (RPIA) and aspartate aminotransferase (GOT) (Supplementary Table S4). Of the 57 quantitative SSs (Supplementary Table S4), 20 SSs were significantly different among the three ‘Anji Baicha’ developmental stages (Fig. 7b). Notably, the succinylation level of the K125 site of PGK (Unigene21236_All) was approximately 3-fold altered in S3vsS1, and approximately 2-fold altered in S2vsS1 and S3vsS2 (Fig. 7; Supplementary Table S4), suggesting that the succinylation of this protein may underlie the periodic albino phenotype.

### SP interaction networks in ‘Anji Baicha’

**Figure 8:**
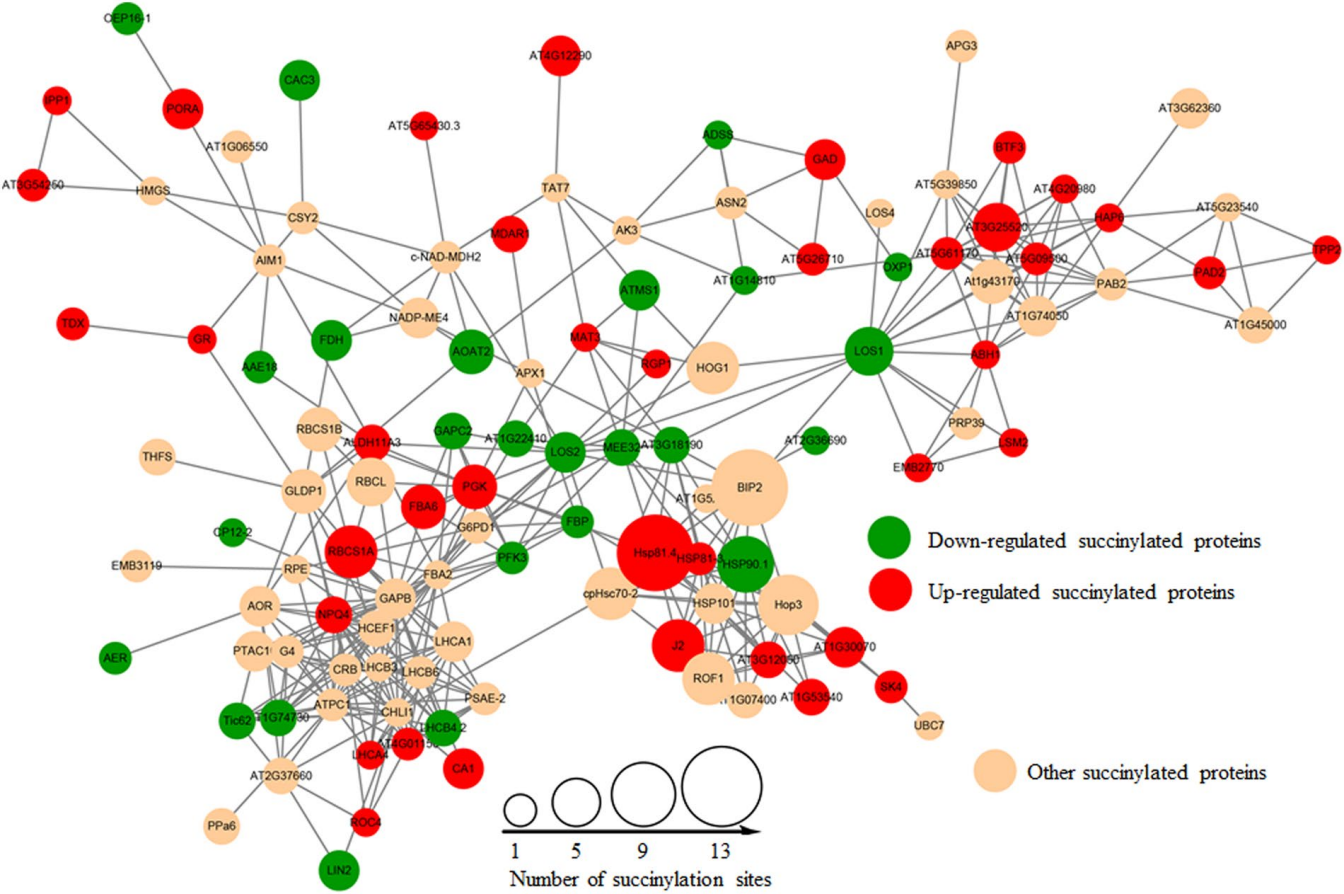
PDSL protein-protein interaction maps in S3vsS2.

The identification of protein-protein interaction networks through bioinformatic analysis is regarded as a useful tool for formulating testable hypotheses to determine the functions of uncharacterized proteins. To further understand the cellular processes regulated by succinylation in ‘Anji Baicha’, we generated protein interaction networks for all PDSLs among the three ‘Anji Baicha’ developmental stages. The protein-protein interaction maps were visualized using Cytoscape software and are shown in Fig. 8 and Supplementary Figs S4 and 5. We identified 385 SPs as network nodes for all PDSLs among the three ‘Anji Baicha’ developmental stages (see Supplementary Table S5).

## Discussion

In this study, we used quantitative proteomics approaches to yield the first extensive data on the lysine succinylome in the tea plant. In total, 3530 SSs corresponding to 2132 SPs were identified in ‘Anji Baicha’ (Supplementary Table S1). A comparison between the three developmental stages of ‘Anji Baicha’ revealed that the PDSLs acted primarily in photosynthesis, carbon fixation, biosynthesis of amino acids, and porphyrin and chlorophyll metabolism (Figs 6 and 7).

A large number of lysine SSs and SPs were recently identified in bacteria such as *M*. *tuberculosis* (1,739 SSs on 686 SPs)^11^ and *E*. *coli* (2,572 SSs on 990 SPs), in the fungus *Saccharomyces cerevisiae* (1,345 SSs on 474 SPs), and in mammals such as *Mouse musculus* (2,140 SSs on 750 SPs) and *Human sapiens* (2,004 SSs on 738 SPs)^9^. These results highlight the importance of lysine succinylation in regulating diverse processes in both prokaryotes and eukaryotes. However, SSs and SPs in plants remain largely uncharacterized. He *et al*.^3^ recently reported the presence of 665 SSs on 261 SPs in rice. Subsequently, Shen *et al*.^18^ identified 325 SSs on 193 SPs in *Taxus*. Here, we identified 3530 SSs corresponding to 2132 SPs in ‘Anji Baicha’ (Supplementary Table S1), representing the first reported succinylome for the tea plant and the largest plant succinylome reported to date. Our study provides a rich database of putative SSs and SPs and provides further confirmation of the widespread and conserved nature of succinylation.

Over the past few decades, studies of the mechanisms underlying leaf coloration have intensified; leaf colour is a useful characteristic for determining the value of horticultural plants and for aesthetics. Albinism has been studied in plant species, including *Arabidopsis*, rice, bean and wheat^33–37^. Transcriptomic and proteomic analyses have identified many albinism-related genes and proteins and have showed a complex network regulating albinism. As an albino tea cultivar, ‘Anji Baicha’ exhibits a high amino acid concentration during the albinistic stage. Leaf colour is not only related to the aesthetics of ‘Anji Baicha’ but is also highly linked to its quality as a consumable. Recently, Li *et al*. (2011, 2015)^22, 31^ identified numerous differentially expressed genes, proteins and metabolites present during periodic albinism in ‘Anji Baicha’. In this study, we focused on protein modification to gain further insight into this mechanism.

Photosynthesis plays a crucial role in metabolism as the source of chemical energy that supports plant life. Previous studies have reported that lysine SPs mapped to the photosynthesis pathway, indicating a potentially conserved function for lysine succinylation in the regulation of photosynthesis^3, 18^. In our research, we identified 66 lysine SSs on 31 SPs related to photosynthesis (PsaB, PsaD, PsaE, PsaF, PsaK, PsaL, PsaN, PsbC, PsbO, PsbP, PsbQ, PsbS, Psb27, LHCA1, LHCA2, LHCA4, LHCB4, LHCB5, LHCB6, PetH, AtpB, AtpD and AtpG), and many quantitative SSs differed significantly among the three ‘Anji Baicha’ developmental stages (Fig. 7a; Supplementary Table S4). Taking into account previous reports of physiological changes during periodic albinism in ‘Anji Baicha’^20–22^, we speculate that there are two possible explanations for the relationship between the difference in succinylation level and the albino phenotype in ‘Anji Baicha’. Abnormal chloroplast development and altered chlorophyll content may affect photosynthetic capacity, thereby altering relevant proteins at the PTM level. Alternatively, a change in the PTM level may affect the expression levels or activities of these proteins, thereby altering photosynthetic capacity and ultimately leading to the albino phenotype.

Our analysis also revealed that proteins involved in the carbon fixation changed significantly (Fig. 7b; Supplementary Table S4). Plants use energy from sunlight to convert carbon dioxide into organic compounds though photosynthesis. During this process, carbon is fixed via the Calvin cycle. A total of 102 SSs on 36 SPs were identified in the carbon fixation photosynthetic pathway (Supplementary Table S4). Interesting, 12 SSs (7 in Unigene9029_All and 5 in CL3466.Contig1_All) in RbcS and 6 SSs (Unigene3523_All) in RbcL were identified, and differential succinylation levels among the three ‘Anji Baicha’ developmental stages were detected for 4 sites (K67 in Unigene3523_All, K144 in Unigene9029_All, K67 and K148 in CL3466.Contig1_All). Rbc has been identified in all photosynthetic organisms examined, and it affects aspects of plant growth and development such as viability, biomass productivity, stress resistance and fecundity. Our study provides further support for a regulatory process of Rbc, as it might play an important role in ‘Anji Baicha’ albinism and other biological process. In addition, the succinylation level of the K125 site of PGK (Unigene21236_All) was approximately 3-fold altered in S3vsS1, and approximately 2-fold altered in S2vsS1 and S3vsS2 (Fig. 7; Supplementary Table S4), suggesting that the succinylation of this protein may underlie the periodic albino phenotype.

## Methods

### Plant material and growth conditions

Tea plants (*Camellia sinensis* cv. ‘Anji Baicha’) were grown under natural conditions and normal horticultural management near the Tea Research Institute of the Chinese Academy of Agricultural Sciences in Hangzhou, China. Healthy leaves from 10-year-old tea plants were sampled for our experiments between April and June 2015.

### Measurement of chlorophyll concentrations

Fresh ‘Anji Baicha’ leaves were collected and weighed, followed by homogenization in an extraction solution composed of acetone:ethanol:water = 4.5:4.5:1 (v/v/v) for 24 h. The chlorophyll content was measured according to Wintermans and De Mots (1965)^38^.

### Protein preparation and trypsin digestion

Protein extraction was performed using the method involving trichloroacetic acid (TCA) combined with acetone. Briefly, ‘Anji Baicha’ leaves were first ground in liquid nitrogen and sonicated in lysis buffer containing 10 mM DTT, 8 M urea, 1% Protease Inhibitor Cocktail, and 2 mM EDTA, pH 7.5. The protein was then precipitated with cold 15% TCA for 2 h at −20 °C, and the precipitate was collected after centrifugation at 5,000 g for 10 min at 4 °C. Finally, the precipitate was washed with cold acetone, and the supernatant was redissolved in 100 mM TEAB with 8 M urea (pH, 8.0). Protein concentration was determined using the 2-D Quant kit according to the manufacturer’s protocol. Protein digestion was performed using a trypsin-based method. The protein solution was reduced in 10 mM DTT for 1 h at 37 °C and alkylated with 20 mM IAA for 45 min at room temperature in darkness. Then, the protein solution was digested with trypsin overnight at 37 °C in a 1:50 trypsin-to-protein mass ratio and a 1:100 trypsin-to-protein mass ratio for the following 4 h.

### Tandem Mass Tag (TMT) labeling and High-Performance Liquid Chromatography (HPLC) fractionation

Both proteome profiling and succinylome profiling were performed with three biological replicates. Three 6-plex TMT reagent kits were respectively used for proteome profiling and succinylome profiling to label the peptide mixture according to the manufacturer’s instructions. Briefly, one unit of TMT reagent (defined as the amount of reagent required to label 100 μg of protein) was thawed and reconstituted in 24 μl acetonitrile. The peptide mixtures were then incubated with the TMT reagent for 2 h at room temperature, pooled, desalted using a Strata X C18 SPE column (Phenomenex) and dried by vacuum centrifugation. The TMT-labeled peptides were fractionated by high pH reverse-phase HPLC using an Agilent 300Extend C18 column (5-μm particles, 4.6 mm ID, 250 mm length). Briefly, the peptides were first separated into 80 fractions and then combined into 6 fractions and dried by vacuum centrifugation.

### Affinity enrichment and LC-MS/MS measurement

To enrich succinylated peptides, the tryptic digestion product was first dissolved in NETN buffer (0.5% NP-40, 50 mM Tris-HCl, 1 mM EDTA and 100 mM NaCl, pH 8.0) and incubated with an anti-succinyl-lysine antibody at 4 °C overnight with gentle shaking. Then, the beads were washed four times with NETN buffer and twice with ddH_2_O. Subsequently, the bound peptides were eluted with 0.1% trifluoroacetic acid and vacuum-dried. The resulting peptides were cleaned with C18 ZipTips (Millipore) according to the manufacturer’s instructions. The peptide mixture was loaded onto a reversed-phase pre-column (Acclaim PepMap 100, Thermo Scientific) and washed with 2% acetonitrile containing 0.1% formic acid. Peptide separation was performed using a reversed-phase analytical column (Acclaim PepMap RSLC, Thermo Scientific). Briefly, for succinylome, the peptide mixture was separated by a linear gradient of 7 to 25% buffer containing 98% acetonitrile and 0.1% formic acid for 20 min, 25 to 40% for 12 min, and increasing to 80% in 4 min then holding at 80% for the last 4 min; and for proteome, the peptide mixture was separated by a linear gradient of 8 to 26% buffer containing 98% acetonitrile and 0.1% formic acid for 22 min, 26 to 40% for 12 min and increasing to 80% in 3 min then holding at 80% for the last 3 min. The flow rate was 400 nl/min. The results were analyzed by Q Exactive^TM^ Plus hybrid quadrupole-Orbitrap mass spectrometer (Thermo Fisher Scientific). The peptides were subjected to an NSI source followed by MS/MS in Q Exactive^TM^ Plus (Thermo) coupled online to the UPLC. Peptides were selected for MS/MS with the NCE setting as 30. Intact peptides and ion fragments were detected in the Orbitrap at resolutions of 70,000 and 17,500, respectively. A data-dependent procedure that alternated between one MS scan and 20 MS/MS scans was applied for the top 20 precursor ions above a threshold ion count of 1E4 in the MS survey scan with 15.0 s (succinylome) and 30.0 s (proteome) dynamic exclusion. Other parameters were as follows: electrospray voltage: 2.0 kV; prevent overfilling of the ion trap: automatic gain control; MS/MS spectra generation: 5E4 ions; m/z scan: 350 to 1800; fixed first mass: 100 m/z.

### Data analysis

MS/MS data were processed using MaxQuant with an integrated Andromeda search engine (v.1.5.2.8). Tandem MS data were searched against the *C*. *sinensis* database (created by our laboratory) concatenated with a reverse decoy database. The detailed parameters used for MaxQuant are as follows. For both of succinylome and proteome, digestion mode: trypisin/P; fixed modification: carbamidomethylation on Cys; max missed cleavages: 4; first search PPM: 20; main search PPM: 5; max charge: 5; max number of modifications per peptide: 5; min razor & unique peptide: 1; min peptide length: 7; peptides for quantification: unique & razor peptides; variable modification: oxidation on Met and acetylation on protein N-terminal. The additional parameters for succinylome: min score for modified peptides: 40; variable modification: succinylation on Lys. False discovery rate thresholds were specified at 1%, and the site localization probability was set as >0.75.

### Bioinformatic analysis

Functional annotation and enrichment analysis were performed using the UniProt-GOA database (http://www.ebi.ac.uk/GOA/) and DAVID Bioinformatics Resources 6.7. Protein pathway annotation was performed using the Kyoto Encyclopedia of Genes and Genomes (KEGG) database^39–42^. Protein domain functional annotation was performed using the InterProScan database. For hierarchical clustering, protein categories that were enriched in at least one of the clusters with a P-value < 0.05 were transformed by the x = −log10 (P-value) function. These x-values were z-transformed and clustered by one-way hierarchical clustering (Euclidean distance, average linkage clustering) in Genesis and visualized by a heat map using the “heatmap.2” function from the “gplots” R-package. Soft motif-x was used to analysis the model of sequences constituted with amino acids in specific positions of modifier-21-mers (10 amino acids upstream and downstream of the site) in all protein sequences. Functional interaction network analysis was performed using the STRING database, and the network was visualized by Cytoscape v2.8.3. Molecular complex detection (MCODE) was utilized to analyze densely connected regions.

### Western Blot

Total protein was extracted from leaves of ‘Anji Baicha’ at the three developmental stages and the extracts were loaded and separated by SDS-PAGE. Then the proteins were transferred onto the PVDF membrane (Millipore). The membrane was incubated in TBST buffer (0.1% Tween 20, 10 mM Tris–Cl, 100 mM NaCl, pH 7.4) containing 5% fat-free milk for 1 h at room temperature, and then washed three times with TBST buffer. Primary antibodies (anti-succinylation, PTM-419, 1:1000 dilution) were diluted in TBST buffer and incubated with the membrane at 4 °C overnight. Then, secondary antibodies (Goat anti-Mouse IgG (H + L), Thermo, 31430) were diluted (1:10000) in TBST buffer and incubated with the membrane at room temperature for 45 min. The membrane blots were incubated in the WesternBrightTM ECL substrate (Advansta) for 1 min lastly and the signal intensities were visualized using the Molecular Image® ChemiDocTM XRS + (Bio-Rad).

## Electronic supplementary material

Supplementary information

Table S1

Table S3

Table S4

Table S5
