# Supplementary material for: Quantitative Succinyl-Proteome Profiling of Camellia sinensis cv. ‘Anji Baicha’ During Periodic Albinism
Source: Sci Rep. 2017 May 12;7:1873. doi: 10.1038/s41598-017-02128-x (PMC5431936; doi:10.1038/s41598-017-02128-x)

Quantitative Succinyl-Proteome Profiling of *Camellia sinensis* cv. 'Anji Baicha' During Periodic Albinism

Yan-Xia Xu[1+], Chen-Jia Shen[2+], Jian-Qiang Ma[1+], Wei Chen[1], Juan Mao[1], Yan-Yan Zhou[3] & Liang Chen[1*]

1. Tea Research Institute of the Chinese Academy of Agricultural Sciences/ Key Laboratory of Tea Biology and Resources Utilization, Ministry of Agriculture, 9 South Meiling Road, Hangzhou 310008, China
2. College of Life and Environmental Sciences, Hangzhou Normal University, Hangzhou 310036, China.
3. Jingjie PTM Biolab (Hangzhou) Co., Ltd., Hangzhou 310018, China

+ These authors contributed equally to this work.

*Corresponding author, E-mail:liangchen@tricaas.com; Tel:+86-571-8665 2835,

Fax:+86-571-8665 0056

Supplementary information files:

Table S1-5 and Figure S1-5.

Supplementary Information

Table S1. Detailed information describing the identified lysine SSs and SPs in 'Anji Baicha' for each of the three developmental stages.

Table S2. Motif analysis of the identified lysine succinylated peptides. K^{su} indicates a succinylated lysine and "." indicates any amino acid.

Motif	Motif Score	Foreground		Background		Fold Increase
		Matches	Size	Matches	Size	
..........K^{su}P.........	16	589	3,148	33,261	718,656	4.04
..........K^{su}.E........	16	495	2,559	46,380	685,395	2.86
.......E..K^{su}K.........	32	132	2,064	6,052	639,015	6.75
.........RK^{su}..........	16	334	1,932	38,499	632,963	2.84
.........KK^{su}..........	16	312	1,598	52,938	594,464	2.19
..........K^{su}K.........	16	256	1,286	41,205	541,526	2.62
..........K^{su}E.........	16	249	1,030	44,929	500,321	2.69
..........K^{su}D.........	16	242	781	31,593	455,392	4.47
..........K^{su}R.........	16	215	539	33,703	423,799	5.02
..........K^{su}.D........	16	106	324	19,530	390,096	6.53
..........K^{su}.P........	16	104	218	18,658	370,566	9.47

Table S3. Detailed information on the proteomic profile of three 'Anji Baicha' developmental stages.

Table S4. The SSs and SPs involved in photosynthesis and carbon fixation in photosynthetic pathways.

Table S5. Information describing the protein-protein interaction network for the PDSLs among three 'Anji Baicha' developmental stages.

Figure S1. (a) Distribution of succinylated peptides according to length in three biological replicates. (b) Distribution of SPs according to the number of SSs in three biological replicates.

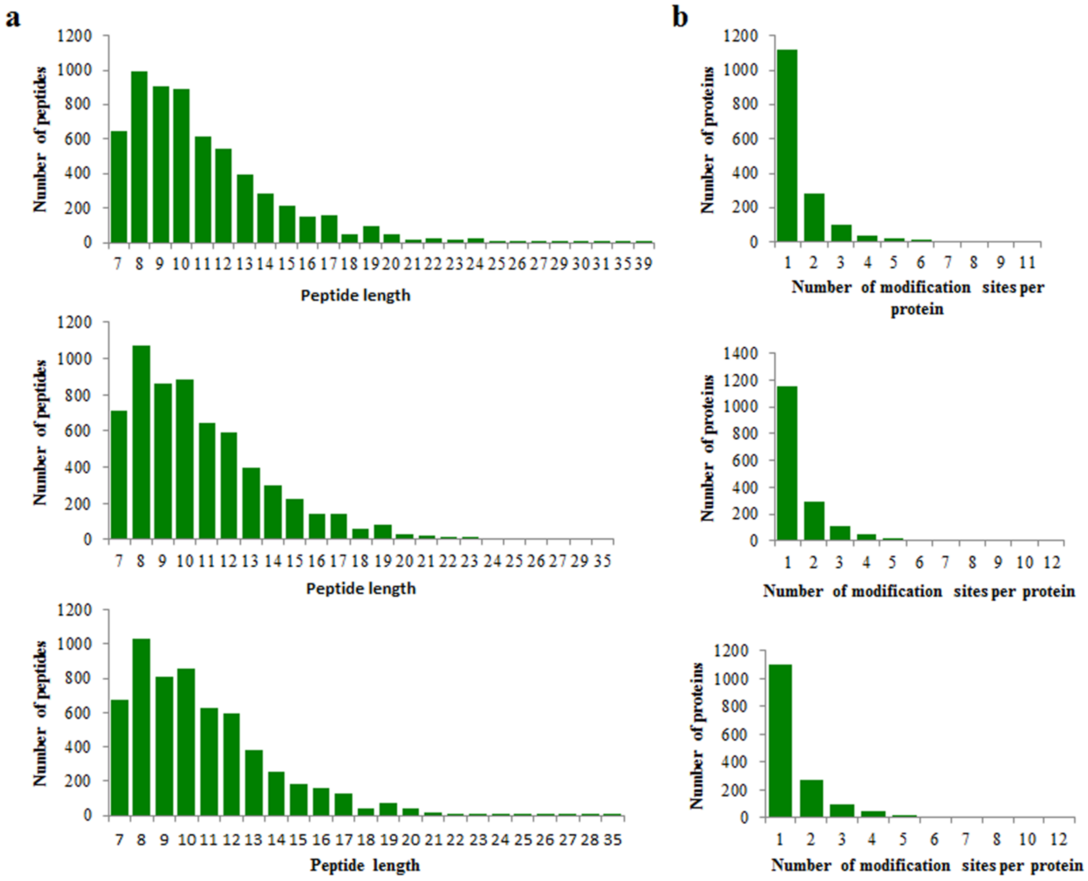

Figure S2. The distribution of mass error for the identified peptides in three biological replicates.

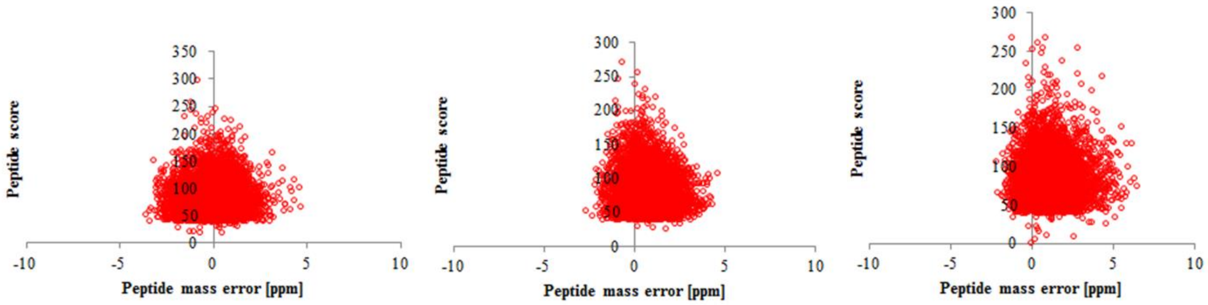

Figure S3. Comparative analysis of differentially succinylated sites and proteins among the three ‘Anji Baicha’ developmental stages.

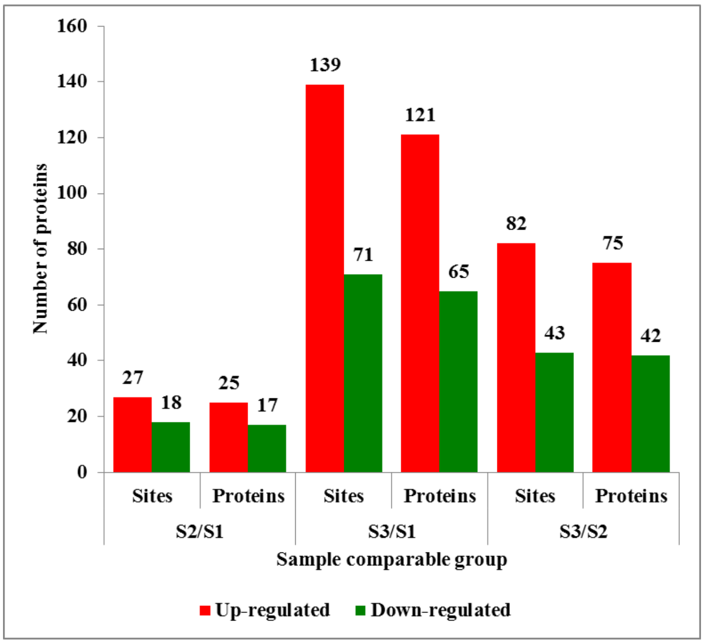

Figure S4. PDSL protein-protein interaction maps in S2vsS1.

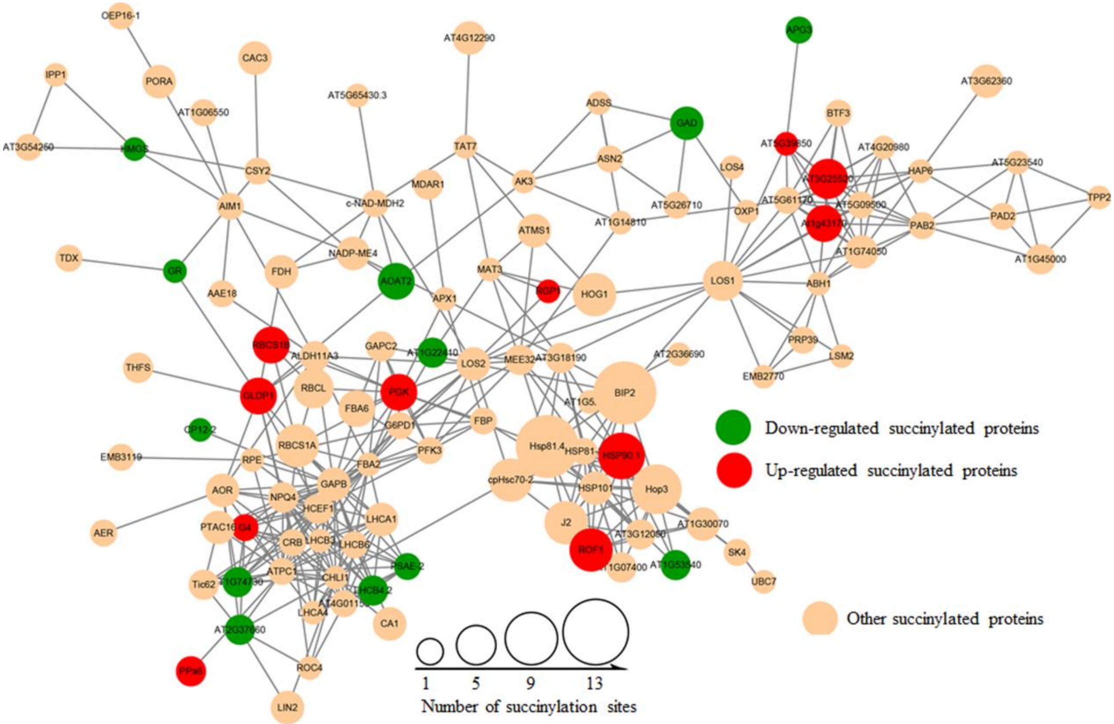

Figure S5. PDSL protein-protein interaction maps in S3vsS1.

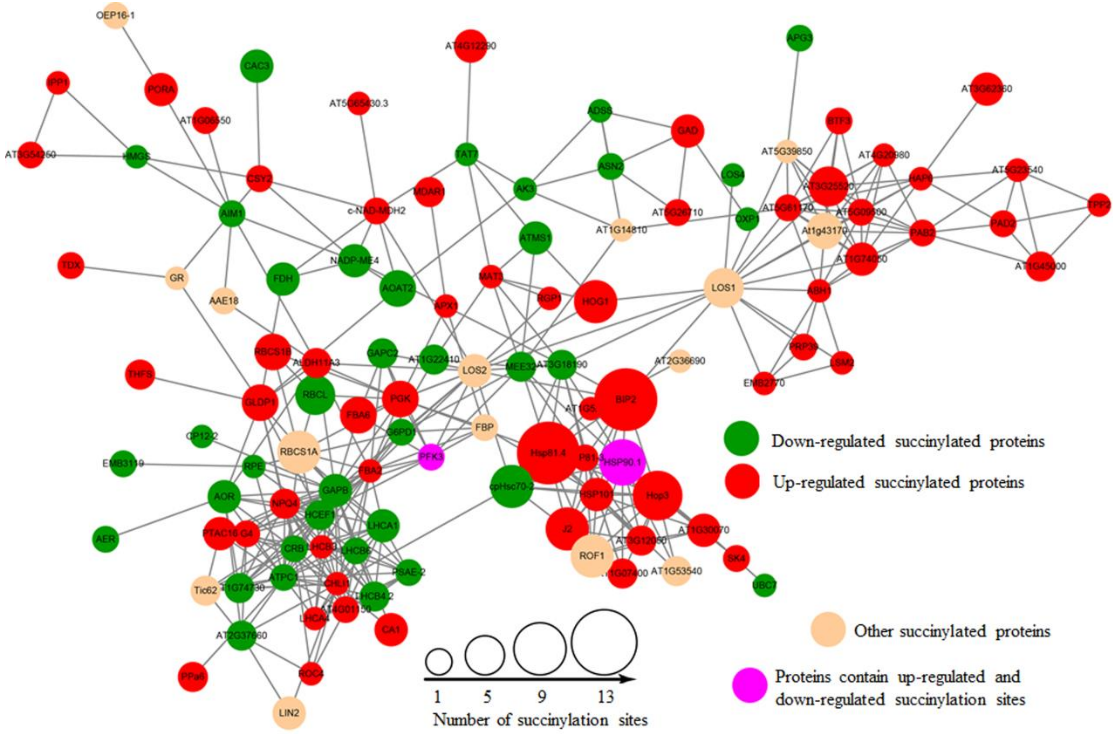

Supplement: Supplementary file 1 — Supplementary information [file 41598_2017_2128_MOESM1_ESM.pdf]
